# Characterization of Side Populations in HNSCC: Highly Invasive, Chemoresistant and Abnormal Wnt Signaling

**DOI:** 10.1371/journal.pone.0011456

**Published:** 2010-07-06

**Authors:** Jun Song, Insoon Chang, Zhuo Chen, Mo Kang, Cun-Yu Wang

**Affiliations:** 1 Laboratory of Molecular Signaling, Division of Oral Biology and Medicine, University of California Los Angeles School of Dentistry, Los Angeles, California, United States of America; 2 Jonsson Comprehensive Cancer Center, University of California Los Angeles, Los Angeles, California, United States of America; 3 Department of Hematology and Oncology, Winship Cancer Institute, Emory University, Georgia, United States of America; 4 Section of Endodontics, Division of Associated Clinical Specialty, University of California Los Angeles School of Dentistry, Los Angeles, California, United States of America; University of Calgary, Canada

## Abstract

Side Population (SP) cells, a subset of Hoechst-low cells, are enriched with stem cells. Originally, SP cells were isolated from bone marrow but recently have been found in various solid tumors and cancer cell lines that are clonogenic *in vitro* and tumorigenic *in vivo*. In this study, SP cells from lymph node metastatic head and neck squamous cell carcinoma (HNSCC) cell lines were examined using flow cytometry and Hoechst 3342 efflux assay. We found that highly metastatic HNSCC cell lines M3a2 and M4e contained more SP cells compared to the low metastatic parental HNSCC cell line 686LN. SP cells in HNSCC were highly invasive *in vitro* and tumorigenic *in vivo* compared to non-SP cells. Furthermore, SP cells highly expressed *ABCG2* and were chemoresistant to Bortezomib and etoposide. Importantly, we found that SP cells in HNSCC had abnormal activation of Wnt/β-catenin signaling as compared to non-SP cells. Together, these findings indicate that SP cells might be a major driving force of head and neck tumor formation and metastasis. The Wnt/β-catenin signaling pathway may be an important target for eliminating cancer stem cells in HNSCC.

## Introduction

HNSCC ranks among the 10 most common cancers worldwide with more than 500,000 new cases diagnosed each year. Despite latest innovations in both basic and clinical research, the overall survival rate for HNSCC still remains low, and it is reported that 25% of patients develop a second cancer within 5 years of diagnosis [1], [2]. Thus, improvement on conventional therapy is urgently needed to effectively target HNSCC. Recently, studies on several solid tumors revealed the existence of a rare subpopulation of tumor-initiating cells, known as “cancer stem cells” (CSCs) [3], [4]. The CSC model of tumor development and progression indicates that CSCs are responsible for tumor initiation, growth, and metastasis [5]. CSCs have the capability to self renew, initiate and maintain tumor growth, and disseminate from the tissue reservoir to promote cancer metastasis [6], [7]. In addition, CSCs exhibit an intrinsic resistance to chemotherapeutic agents, preventing complete elimination of the tumor. Therefore, understanding properties and mechanisms of CSCs as a molecular target is essential to develop effective anti-cancer therapy against tumorigenesis [8], [9].

Side population (SP) cells are a subset of enriched progenitor cells exhibiting CSC-like phenotypes with a distinct low Hoechst 33342 dye staining pattern [10], [11]. SP cells have been identified and isolated from various solid tumors, highly express stem cell markers, and exhibit the ability to self-renew as well as give rise to differentiated tissue cells [10], [12]. Florescent dye exclusion of SP phenotype results from the expression of ATP-binding cassette (ABC) family transporter proteins such as ABCG2 in cultured human mammary epithelial cells. Chemotherapeutic resistance of SP cells against conventional anticancer drugs is thought to be associated with high ABCG2 expression [13], [14]. Furthermore, SP cells demonstrated elevated functional progenitor activity compared to non-SP cells, signifying that the accumulation of SP cells enhances the risk of tumor development [15]–[18].

Based on the CSC model of tumor development and progression, in this study, we hypothesized that SP cells might be enriched in metastatic HNSCC cell lines. 686LN is a HNSCC cell line established from human lymph node metastasis, and M3a2 and M4e are high metastatic cell lines derived from a low metastatic 686LN cell line through several *in vivo* selections [19], [20]. We found that high metastatic M3a2 and M4e cell lines contain significantly higher quantity of SP cells compared to the low metastatic 686LN cell line. Purified fraction of SP cells in HNSCC exhibited resistance to chemotherapeutic agents such as Bortezomib and etoposide, attributed to high expression of ABCG2. Moreover, compared to non-SP cells, SP cells were highly invasive and had abnormal activation of Wnt/β-catenin signaling [21]. Together, these findings indicate that SP cells might be a major driving force of head and neck tumor progression and metastasis. The Wnt/β-catenin signaling pathway may be an important target for eliminating CSCs in HNSCC.

## Materials and Methods

### Cell culture and Retroviral infection

The HNSCC cell lines 686LN, M3a2 and M4e were maintained as a monolayer culture in Dulbecco's modified Eagle's medium (DMEM)/F12 medium (1∶1) supplemented with 10% fetal bovine serum (FBS) (Invitrogen, Carlsbad, CA). To eliminate the possible effect of Hoechst 33342 dye on cell viability, sorted SP and non-SP cells from M3a2 and M4e cells were incubated in culture medium at 37°C for 24 hours to recover from Hoechst staining. After 24 hours, cells were detached and plated for experimentation. For cytotoxicity assay, cells were treated with PS-341 (0.5 µM) or etoposide (20 µM) for 24 and 48 hr, and cell viability was determined using Trypan blue exclusion assay.

Human ABCG2 cDNA was purchased from ATCC (GenBank accession No. BC021281). PCR was performed to obtain the HA-tagged ABCG2 cDNA using a specific set of primers (5′-ATGGATCCGCTCCCATCGTGACCTCCAG-3′ and 5′-ATGAATTCTTA CGCATAGTCAGGAACATCGTATGGGTAAGAATATTTTTTAAGAAAT-3′. The PCR product was purified and subcloned into XhoI and EcoRI sites of the retroviral pQCXIP vector (Clontech, Mountain View, CA). Retroviruses were packaged in 293T cells as described previously [22]. Cells were infected with retroviruses expressing HA-tagged ABCG2 or empty vector and selected with puromycin (2 µg/ml) for 2 weeks.

### Flow cytometry, immunostaining and Western blot analysis

Cells were washed with PBS, detached from the culture plate with trypsin and EDTA, pelleted, and resuspended in 37°C DMEM containing 2% FBS at 1×10^6^ cells/ml. The cells were incubated with Hoechst 33342 (Sigma, St. Louis, MO) at 5 µg/ml either alone or in combination with ABC transporter inhibitors verapamil (50 µM) or reserpine (20 µM, Sigma) for 90 min at 37°C. After staining, the cells were centrifuged and resuspended in HBSS (Invitrogen) containing 1 µg/ml propidium iodide and maintained at 4°C for flow cytometry analysis and sorting. Cell sorting was performed on a FACSVantage SE (Beckton Dickson, Mountain View, CA). The Hoechst 33342 emission was first split by using a 610-nm dichroic short-pass filter, and the red and the blue emissions were collected through 670/30- and 450/65-nm bandpass filters, respectively. The parental M3a2 and M4e cells without Hoechst 33342 staining were also collected under the same condition. For surface marker analysis, cells stained with Hoechst 33242 were further incubated (30 min at 4°C) with anti-CD29-APC (1∶100), or anti-CD44-APC (1∶100; BD PharMingen) and then stained with propidium iodide (1 µg/ml) before analysis. For Western blot analysis, whole cell lysates were extracted and separated on 10% SDS-PAGE as described previously [22]. Primary antibodies rabbit anti-HA (Covance) and mouse anti-tubulin were purchased from Santa Cruz Biotechnology Inc. and secondary antibodies mouse anti-rabbit-IgG-horseradish peroxidase (HRP) and goat anti-mouse-IgG-HRP were purchased from Promega. The signals were detected using ECL reagents (Pierce, Rockford, IL, USA).

### Reverse transcriptase PCR (RT-PCR) and Real-time RT-PCR

Total RNA was isolated from cells using Trizol solution (Invitrogen), followed by first strand cDNA syhthesis using SuperScript First-Strand Synthesis System for RT-PCR (Invitrogen) as directed by the manufacturer's instruction. The primers for PCR are as follow: *BMI 1*, 5′-CTCCCAACTGGTTCGACCTT-3′, and 5′-CGGTTTCCATATTTCTCAGT-3′; *p63*, 5′-CTTTGCTGAGGGTTTGAATA-3′, and 5′-CTAGTGGTTTCTATGCTTAC-3′; *ABCG2*, 5′-ACACAAAAGCCTACTCAGCC-3′, and 5′-GTCAATCTAAAATTATTTCC-3′; *ABCB5*, 5′-CACAAGTTGGACTGAAAGGA-3′, and 5′-ACCACTAGGCATGTCCTTCC-3′; *MDR1*, 5′-ACAGGAAGAGATTGTGAGGG-3′, and 5′-TATCCAGAGCTGACGTGGCT-3′; *AXIN2*, 5′-CTGGCTTTGGTGAACTGTTG-3′, and 5′-AGTTGCTCACAGCCAAGACA-3′; *DKK1*, 5′-AGCACCTTGGATGGGTATTC-3′, and 5′-CACAATCCTGAGGCACAGTC-3′. RT-PCR and Real-time RT-PCR were performed using Platium Taq polymerase (Invitrogen). The RT-PCR cycle conditions were 95°C for 2 min followed by 30 cycles of 95°C for 15 sec, 60°C for 30 sec and 72°C for 30 sec. Real-time RT-PCR was performed using an iCycler iQ Real-time PCR detection system (Bio-Rad). The PCR cycle conditions were 95°C for 2 min followed by 45 cycles of 95°C for 15 sec and 60°C for 30 sec. All samples were run in triplicates in the same culture plate.

### Soft agar assay and sphere assay

A six-well culture plate was coated with 2 ml bottom agar mixture (DMEM/F12 with 10% FBS, 0.6% agar). After the bottom layer was solidified, 2 ml top agar-medium mixture (DMEM/F12 with 10% FBS, 0.3% agar) containing 2×10^4^ cells was added, and the plate was incubated at 37°C for 3 weeks. Plate was stained with 0.005% crystal violet, and the colonies were counted.

The sphere assay was adopted from a previously published protocol [20]. Sorted SP and non-SP cells were resuspended in 1∶1 Matrigel (BD Biosciences)/serum-free DMEM/F-12 containing human recombinant basic fibroblast growth factor (bFGF; 10 ng/mL), and epidermal growth factor (EGF, 10 ng/mL) in a total volume of 100 µl. Samples were plated around the rims of wells in a 12-well plate and allowed to solidify at 37°C for 10 minutes, before 1 ml of serum-free DMEM/F-12 containing bFGF and EGF was added. Medium was replenished every 3 days. Two weeks after plating, spheres with a diameter over 40 µm were counted.

### Matrigel invasion assay

The invasive ability of sorted SP and non-SP cells was determined using 12- or 24-well Matrigel invasion chambers (BD Biosciences Discovery Labware). Cells were seeded into upper inserts at 0.5×10^5^ (24-well) or 2×10^5^ (12-well) per insert in serum-free DMEM. Outer wells were filled with DMEM containing 5% FBS as chemoattractant. Cells were incubated for 48 hrs, and then non-invading cells were removed by swabbing top layer of Matrigel with a cotton swap. Membranes containing invading cells were stained with a HEMA-3 kit (Fisher). The invading cells on the entire membrane were counted under light microscope.

### Luciferase assay

1×10^6^ cells were seeded into 12-well plates and were transfected with 100 ng of TOPFLASH or FOPFLASH using Lipofectamine 2000 (Invitrogen) according to the manufacturer's protocol. 20 ng of pCMV-RL were co-transfected as an internal control. Cell lysates were collected 24 hr post-transfection and the luciferase activity was measured using the Dual-Luciferase Reporter Assay System (Promega, Madison, WI, USA) according to manufacturer's protocol.

### *In vivo* tumorigenicity

All animal practices in this study were performed in accordance with the institutional animal welfare guidelines of the university. A variety of sorted SP and non-SP cells (ranging from 10^2^ to 10^6^) were resuspended in 100 µL of a DMEM/Matrigel (Matrigel Basement Membrane Matrix; Becton Dickinson) mixture (1∶1 v/v) and injected subcutaneously into 6-week-old nude mice (Jackson Laboratory). Groups of mice were inoculated with SP cells at 1×10^5^, 1×10^4^, 1×10^3^, and 1×10^2^ or non-SP cells at 1×10^6,^ 1×10^5^, 1×10^4^, and 1×10^3^. Tumor growth was monitored for 8 weeks, after which they were euthanized and tumor formation was assessed. The tumor volume was calculated according to the formula: (length × width^2^)/2.

### Statistical methods

Microsoft Office Excel 2003 and the statistical software SPSS12.0 were used in data processing and in analyzing the significance between SP and non-SP cells with the unpaired or paired *t* test. *P* values <0.05 were considered significant. Data were expressed as the mean ± SD from three independent experiments.

## Results

### SP cells exist in highly metastatic HNSCC cells

**Figure 1:**
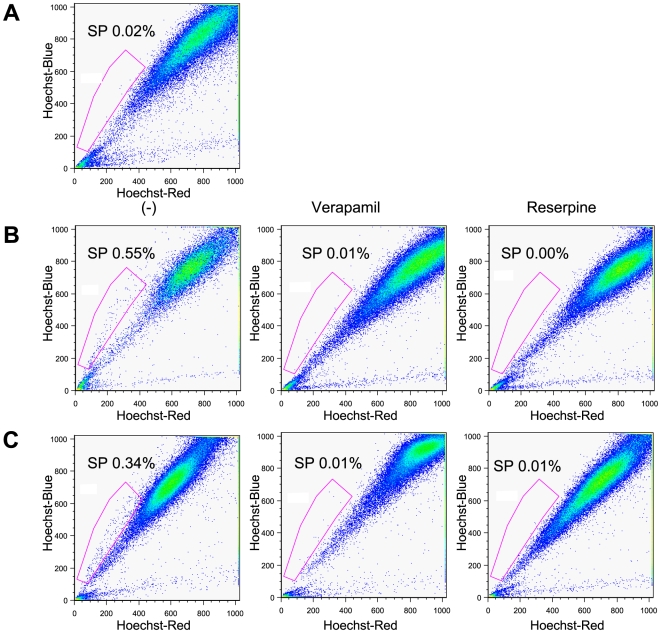
SP cells in high metastatic M3a2 and M4e cells. (**A**): Flow cytometry analysis of 686LN cells. Cells were stained with Hoechst 33342. (**B and C**): M3a2, and M4e cells were stained with Hoechst 33342 in the absence or presence of either verapamil or reserpine. The SP cells were gated and shown as percentage as indicated. Flow cytometry analyses were performed in triplicate with similar results in each case.

SP cells have been shown to exhibit distinct projection pattern by actively effluxing Hoechst 33342 dye from the cytoplasm through verapamil-sensitive ABC transporters. To identify and purify SP cells from the lymph node metastatic HNSCC cell lines, we used fluorescent dye Hoechst 33342 and FACS sorting. Flow cytometric analysis of 686LN, M3a2, and M4e cells stained with Hoechst 33342 are shown (Fig. 1). For low metastatic parental cell line 686LN, 0.02% of the total population was comprised of SP cells (Fig. 1A), whereas SP cells represented 0.55% and 0.34% of the population of M3a2 and M4e cells (Fig. 1B and C), respectively. The level of SP cell population was substantially diminished in the presence of verapamil or reserpine, inhibitor of ABC transporter, in both M3a2 and M4e cells (Fig. 1B and C). Our results suggest that significantly higher population of SP cells exists within the highly metastatic cell line in comparison to the low metastatic cell line.

**Figure 2:**
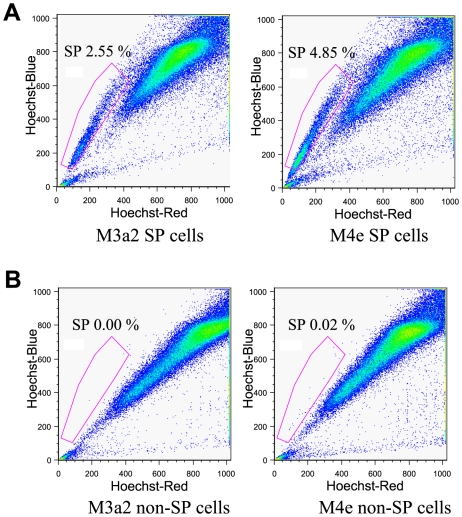
Re-sorting of SP and non-SP cells. (**A**): Cultured SP cells divide into both SP and non-SP cells. (**B**): non-SP cells only divide into non-SP cells. Flow cytometry analyses were performed in triplicate with similar results in each case.

Isolated SP cells and non-SP cells of M3a2 and M4e cell lines were cultured separately under the same pre-sorting conditions for 2 weeks. Afterwards, cultured SP cells and non-SP cells were sorted again by flow cytometry with Hoechst 33342 dye to reanalyze the SP proportion. Intriguingly, re-sorting results demonstrated that SP cells of M3a2 and M4e were able to generate both SP and non-SP cells (Fig. 2A). Conversely, non-SP cells solely repopulated into non-SP cells (Fig. 2B). In addition, population of cells generated from originally isolated SP cells comprised higher fractions of SP cells. The initial percentage of SP cells from first isolation of M3a2 and M4e cells were 0.55% and 0.34%, respectively; however, after re-sorting, the percentage of SP cells were 2.55% for M3a2 and 4.85% for M4e cells.

### Expression of stem cell markers and ABC transporter genes

**Figure 3:**
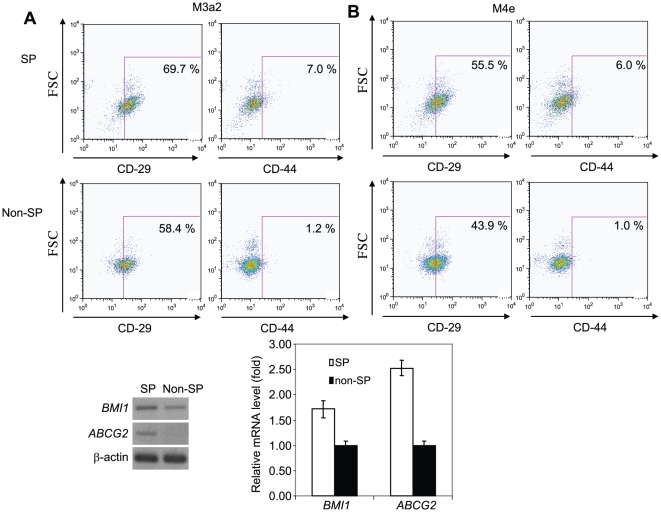
Expression of stem cell markers in SP and Non-SP cells. (**A**): The expression of CD29 and CD44 in SP and non-SP cells from M3a2 cells. Cells were stained with Hoechst 33342 in the presence of either CD29-APC or CD44-APC. Positive CD29 and CD44 were indicated as percentages in both SP and non-SP cells. (**B**): The expression of CD29 and CD44 in SP and non-SP cells from M4e cells. (**C**): Detection of BMI1 and ABCG2 in SP and Non-SP cells by RT-PCR. (**D**): Detection of BMI1 and ABCG2 in SP and non-SP cells by Real-time RT-PCR.

To determine stem phenotype of SP cells, we next examined the expression of two previously reported stem cell surface markers, CD29 and CD44 in SP and non-SP cells [4], [22], [23]. There was no big difference in CD29 and CD44 expression between SP and non-SP cells. For M3a2 cell line, 69.7% of SP cells and 58.4% of non-SP cells were CD29 positive, and 7.0% of SP cells and 1.2% of non-SP cells were CD44 positive (Fig. 3A). For M4e cell line, 55.5% of SP cells and 43.9% of non-SP cells were CD29 positive, and 6.0% of SP cells and 1.0% of non-SP cells were CD44 positive (Fig. 3B). To further investigate the stem phenotype of SP cells, we examined the expression of other stem cell markers including BMI 1, p63 and ABC transporter genes (*ABCG2, ABCG5*, and *MDR1*) using RT-PCR. While the expression of *BMI 1* was marginally increased in SP cells compared to non-SP cells, *ABCG2* were detected in SP cells but not in non-SP cells (Fig. 3C). There was no difference in the expression of *p63*, *ABCG5* and *MDR1* between SP and non-SP cells (data not shown). Moreover, Real-time RT-PCR confirmed that the expression of *ABCG2*, but not *BMI 1*, was significantly higher in SP cells than that in non-SP cells (Fig. 3C).

### SP cells have higher tumor initiation ability *in vitro* and *in vivo*

**Figure 4:**
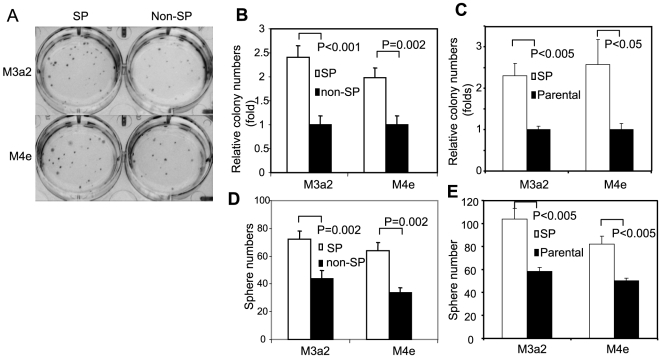
Anchorage-independent growth of SP and non-SP cells. (**A**): Soft agar assay using SP and non-SP cells from M3a2 and M4e. (**B**): Quantification of soft agar assay of SP and non-SP cells. Relative colony numbers (fold) were indicated. (**C**): Soft agar assay using SP cells and their parental cells. Relative colony numbers (fold) were indicated. (**D**): Sphere assay of SP and non-SP cells. The sphere numbers were counted. (E): Sphere assay of SP cells and their parental cells. The sphere numbers were counted.

To minimize the non-specific effects of Hoechst dye on non-SP cells, we cultured both SP and non-SP cells for 24 hr to remove dead cells and then performed all experiments described below. We found that the proliferation rate of SP cells was similar to that of non-SP cells and parental HNSCC cells (Fig. S1). To explore the difference in clonogenic ability between SP and non-SP cells *in vitro*, we performed a soft agar assay. SP cells from M3a2 cells were able to form 2.5 times more colonies than non-SP cells (Fig. 4A and B). Similarly, M4e SP cells formed twice as many colonies than its non-SP counterparts (Fig. 4A and B). To further rule out side effect of Hoechst dye on non-SP cells, we also passed parental M3a2 and M4e cells through flow cytometry without Hoechst dye incubation. A soft agar assay confirmed that SP cells formed notably more colonies than its parental M3a2 and M4e cells (Fig. 4C). In addition to soft agar assay, we performed sphere assay, showing that SP cells have a higher capability in forming spheres compared to non-SP cells and parental cells (Fig. 4D and E). Moreover, the spheres from SP cells, but not non-SP cells, could be passaged in vitro (Fig. S2). However, it should be mentioned that the spheres from tertiary passages was smaller than from first and secondary passages.

Next, we investigated the *in vivo* tumor formation potential of the SP cells. Various numbers of SP and non-SP cells (ranging from 1×10^2^ to 1×10^6^ cells) were injected into 6-week-old nude mice subcutaneously and tumor growth was monitored for 8 weeks. 1×10^2^ SP cells were able to form tumors whereas significantly higher amount of non-SP cells (1×10^4^ cells) were required to initiate tumor formation (Table 1). Furthermore, we found out that mean volume of tumors formed from SP cells were larger than from non-SP cells. With 1×10^4^ cells, SP cells formed a tumor with a mean volume of 41.7 mm^3^ while non-SP cells formed tumor with 6.0 mm^3^ mean volume. With 110^5^ cells, SP cells formed a tumor with a mean volume of 156.4 mm^3^ while non-SP cells formed tumor with 29 mm^3^ mean volume. Taken together, these data indicate that SP cells may contribute to the malignancy of HNSCC cells.

**Table 1:** Tumor formation of SP and non-SP cells.

	Cells injected	Mice with tumors	Average tumor volume (mm^3^)
SP	10^5^	7/7	156.4±101.3
	10^4^	6/7	41.7±43.6
	10^3^	4/7	13.9±5.6
	10^2^	2/7	7.0±1.4
Non-SP	10^6^	7/7	107.1±70
	10^5^	6/7	29.0±23
	10^4^	2/7	6.0±0
	10^3^	0/7	0.0

### Increased invasion ability of SP cells

**Figure 5:**
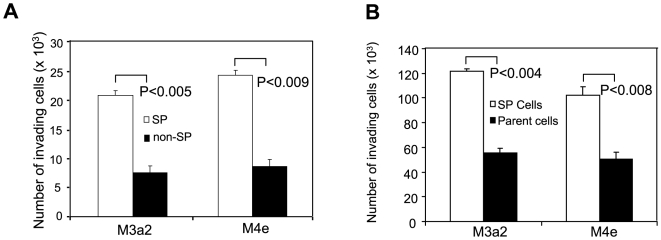
SP cells are highly invasive. (**A**): Invasive growth of SP and non-SP cells. The invasion assay was performed using 24-well Matrigel invasion chambers. The results represent average value ± SD from three independent experiments. (**B**): Invasive growth of SP cells and their parental cells. The invasion assay was performed using 12-well Matrigel invasion chambers.

Since highly metastatic HNSCC cell lines M3a2 and M4e contain more SP cells compared to the low metastatic parental HNSCC cell line 686LN, we hypothesized that SP cells might be more invasive than non-SP cells. The invasion ability of SP and non-SP cells was examined by Matrigel invasion assay. We found SP cells from M3a2 and M4e were significantly more invasive than non-SP cells in Matrigel (Fig. 5A). Moreover, to rule out non-specific effects of Hoechst dye on non-SP cells, we also compared the invasive ability between SP cells and parental HNSCC cells. We found SP cells from M3a2 cells and M4e cells were also more invasive than its respective parental cells (Fig. 5B).

### SP cell chemoresistance

**Figure 6:**
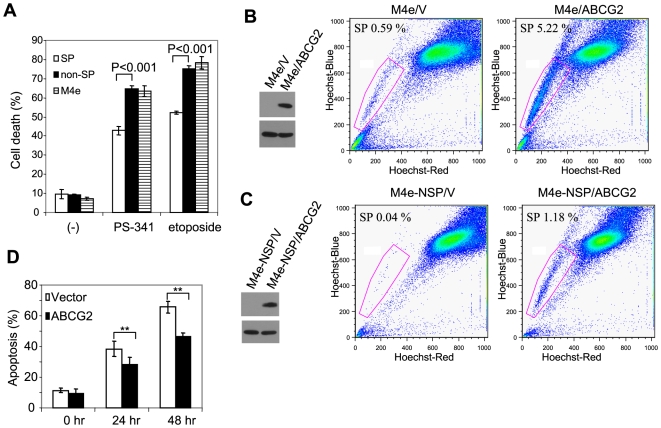
SP cells are resistant to chemotherapy. (**A**): Treatment of SP cells, non-SP cells and the parental M4e cells with PS-341 and etoposide for 48 hr. Cells were counted using Trypan blue exclusion assay. (**B**): Overexpression of ABCG2 increases SP cell numbers. M4e cells were transduced with retroviruses expressing ABCG2 and empty vector. The expression of the HA-tagged ABCG2 was confirmed by Western blot analysis (left panel). M4e/V and M4e/ABCG2 cells were stained with Hoechst and sorting. (**C**): ABCG2 switched non-SP cells to SP cells. Non-SP cells from M4e were transduced with retroviruses expressing ABCG2 or the empty vector. M4e-NSP/V and M4e-NSP/ABCG2 cells were stained with Hoechst and sorting. (**D**): ABCG2 promoted chemoresistance. M4e-NSP/V and M4e-NSP/ABCG2 cells were treated with PS-341 for 24 and 48 hr.

Previous studies on SP cells revealed that SP cells possess chemo-resistance ability against anticancer drugs and ABCG2 transporter is responsible for this resistance phenotype [13], [14]. To exclude the possible toxic effect of Hoechst 3342 on non-SP cells which might affect our results, both SP cells and non-SP cells were plated in 6-well plates for 24 hr to allow them to recover, and the attached cells were subsequently harvested and re-plated for our assay. Of note, we found that Hoechst dye had modest cyotoxic effects on non-SP cells 24 hr after staining. However, after the recovery, there was no difference in cell viability between SP and non-SP cells (Fig. S1). To determine the resistance ability of SP cells, we treated SP and non-SP cells with proteasome inhibitor Bortezomib (also known as PS-341) and etoposide. Trypan blue exclusion assay showed that SP cells had significantly higher survival rate than non-SP cells (Fig. 6A). Addition of reserpine to SP cells partially restored sensitivity of the SP cells to Bortezomib and etoposide (data not shown). Moreover, we found that SP cells were more resistant to Bortezomib and etoposide than parental M4e cells (Fig. 6A), confirming that SP cells are more resistant to chemotherapeutic drugs.

To further investigate the role of ABCG2 in chemoresistance, we generated a M4e cell line stably expressing HA-tagged ABCG2 using retroviral infection (Fig. 6B). ABCG2 expressing cells (M4e/ABCG2) and control cells expressing empty vector (M4e/V) were stained with Hoechst 33342 dye and analyzed with flow cytometry. M4e/V comprised 0.59% of SP cells (Fig. 6B), which was similar to the parental M4e cells; however, M4e/ABCG2 cells contained 5.22% SP cells, indicating that ABCG2 expression is associated with the Hoechst 3342 efflux and SP phenotype. Additionally, we examined whether over-expression of ABCG2 in non-SP cells would allow non-SP cells to acquire SP phenotype. Non-SP cells from M4e were transduced with retroviruses expressing HA-tagged ABCG2 or empty vector (Fig. 6C). Flow cytometry analysis showed that the over-expression of ABCG2 was able to convert non-SP cells into SP cells (Fig. 6C). Finally, we tested whether over-expression of ABCG2 promoted chemoresistance. Both M4e-NSP/ABCG2 and M4e-NSP/V cells were treated with PS-341 for 24 hr and 48 hr, and the result demonstrated that the survival rate of non-SP cells over-expressing ABCG2 was significantly higher compared to the control cells (Fig. 6D).

### Abnormal activation of Wnt/β-catenin signaling in SP cells

**Figure 7:**
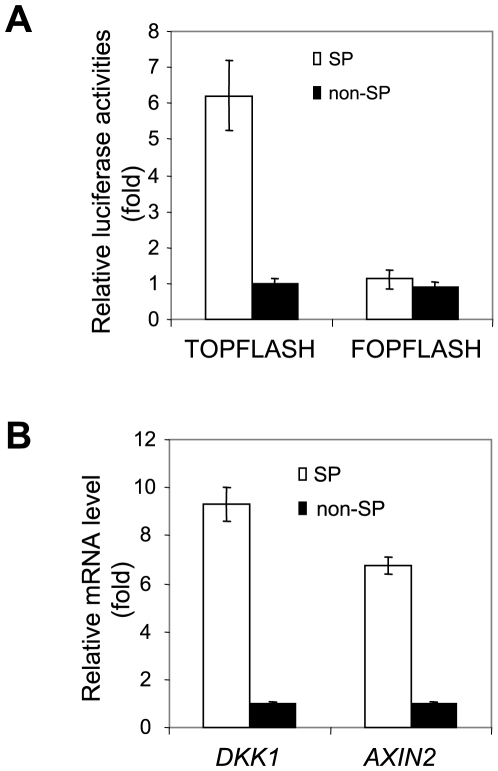
Abnormal activation of Wnt/β-catenin signaling in SP cells. (**A**): Increased Wnt/β-catenin activity in SP cells. SP and non-SP cells were transfected with TOPFLASH or FOPFLASH luciferase reporter assay. Relative luciferase activities were determined. (**B**): Endogenous Wnt target genes were highly expressed in SP cells. The expression of DKK1 and Axin2 was determined using Real-time RT-PCR. Relative mRNA expression levels were determined.

Since the Wnt/β-catenin signaling pathway has been found to be associated with stem cell property or self-renewal, we explored whether canonical Wnt signaling is activated in SP cells using TOPFLASH and FOPFLASH luciferase reporter assay. TOPFLASH reporter contains wild type β-catenin binding sites and its activity is correlated with Wnt/β-catenin-mediated gene transactivation, and mutated β-catenin binding sites which are incorporated into FOPFLASH reporter as a negative control. The TOPFLASH reporter revealed that the activity of β-catenin-dependent transcription was significantly elevated in SP cells compared to non-SP cells (Fig. 7A). To further confirm our results, we examined the expression of two known Wnt/β-catenin target genes, *DKK1* and *AXIN2*. Real-time RT-PCR found that expression of both *DKK1* and *AXIN2* was significantly higher in SP cells (Fig. 7B). Our results suggest that a possible correlation may exist between activated Wnt/β-catenin signaling and the SP cell phenotype.

## Discussion

Since the existence of ‘stem-like’ cells within a heterogeneous mixture of cells comprising an acute myeloid leukemia has been discovered, extensive research has been done on this rare subset of tumor cells, referred as CSCs, to investigate its role in tumorigenesis [24], [25]. CSCs exhibit an ability to undergo self-renewal while sustaining a multipotent differentiation capacity to maintain tumor development indefinitely [3], [5]. In addition, CSCs display resistance to anticancer reagents, preventing the complete elimination through a conventional chemotherapy approach [8]. Recent research on various solid tumors revealed the existence of CSCs, providing strong evidence for the presence of functional heterogeneity within the tumor population [26]. The establishment of highly metastatic cell lines, M3a2 and M4e, which are derived from a poorly metastatic parental cell line, 686LN, in a mouse model of lymph node metastatic, suggest that such intratumor heterogeneity may exist within HNSCC [19], [20]. Side population (SP) cells are a rare subset of cells enriched with progenitor cells that exhibit a distinct low Hoechst 33342 dye staining pattern [10]. SP cells express distinct cancer stem cell like properties and are clonogenic *in vitro* and tumorigenic *in vivo* [12]. Based on Hoechst 33342 efflux ability of SP cells, we have successfully identified and isolated SP cells from highly metastatic M3a2 and M4e cell lines. Moreover, we found that the high metastatic cell line contained significantly high percentage of SP cells compared to the low metastatic cell line. SP cells from HNSCC were highly tumorigenic, invasive and chemoresistant. Our results suggest that SP cells may play a critical role in HNSCC progression and metastasis.

According to the CSC model, CSCs have ability to repopulate tumors even when the majority of non-tumorigenic cancer cells were killed by chemotherapy and radiotherapy [8]. Recent studies suggest that the *ABCG2* transporter plays an important role in SP cell chemo-resistance [27], [28]. Our RT-PCR results confirmed that ABCG2 was expressed in SP cells but not in non-SP cells. To confirm the role of ABCG2 in SP Hoechst 33342 efflux phenotype and chemoresistance in HNSCC, we over-expressed ABCG2 in non-SP cells and found that the over-expression of ABCG2 could promote non-SP cells to acquire SP Hoechst 33342 efflux phenotype and chemoresistance. However, it should be pointed out that over-expression of ABCG2 was unable to fully convert non-SP cells into be SP cells. It suggests that additional factors may control the phenotypes of SP cells. Further studies are also needed to examine whether ABCG2 expression correlates to other phenotypes of SP cells such as tumorigenesis.

Our *in vivo* study revealed that significantly lower numbers of SP cells were required to initiate tumor formation when compared with non-SP cells, and SP cells formed larger tumors at a higher frequency than that of non-SP cells. Both *in vitro* and *in vivo* study on SP cells suggested that the malignancy of M3a2 and M4e cells was largely dependent on the SP cells. In theory, CSCs in solid tumors are source of tumor metastasis [15], [16], [18]. Because of different tumorigencity, we were unable to compare metastatic potentials between SP and non-SP cells in vivo. However, our in vitro invasion assay found that SP cells were at least twice more invasive than non-SP cells. Since larger subpopulation of SP cells existed within highly metastatic cell lines, M3a2 and M4e, our results suggest that SP cells may be the major driving force of HNSCC metastasis.

To further characterize SP cells in HNSCC, we examined the expression of three previously reported stem cell markers, BMI 1, CD29 and CD44 in HNSCC [4], [22], [23]. Despite the difference in tumorigenecity between SP and non-SP cells, these stem cell markers were not dramatically altered between SP and non-SP cells. Importantly, we found that Wnt/β-catenin activation was notably increased in SP cells compared to non-SP cells. Previously, our studies have demonstrated that abnormal activation of Wnt signaling in HNSCC promotes invasive growth of SCC cells and provides resistance to chemotherapeutic drugs [29]–[31]. Our results suggest that abnormal activation of Wnt signaling may contribute to the phenotypes of SP cells, and targeting Wnt signaling may help to eliminate SP cells in HNSCC. Further investigation on mechanisms involved in the activation of Wnt/β-catenin pathway in SP cells may provide an effective model to study the molecular basis of CSCs and to develop targeted anti-cancer therapies.

## Supporting Information

Figure S1 SP cells proliferate at the same rate as non-SP cells and parental HNSCC cells. (A): Both SP cells and parental HNSCC cells were grown in plates for 24, 48 and 72 hrs and cell numbers were counted. (B): Both SP and non-SP cells were grown in plates for 24, 48 and 72 hrs and cell numbers were counted. (C): Both SP and non-SP cells were cultured in plates for 24 hr and cell viability was determined. Afterwards, cells were re-cultured for additional 24 (48 hr time point) or 48 hr (72 hr time point) and cell viability was determined, respectively.
(0.01 MB PDF)

Figure S2 The spheres from SP cells, but not from non-SP cells, could be passaged. Sorted SP and non-SP cells were plated in 12-well plates for 2 weeks. The cells from spheres were harvested and replated every two weeks. The spheres from SP cells could be passaged.
(3.19 MB EPS)
