# Supplementary figures and images for: Characterization of Side Populations in HNSCC: Highly Invasive, Chemoresistant and Abnormal Wnt Signaling
Source: PLoS One. 2010 Jul 6;5(7):e11456. doi: 10.1371/journal.pone.0011456 (PMC2897893; doi:10.1371/journal.pone.0011456)

**A**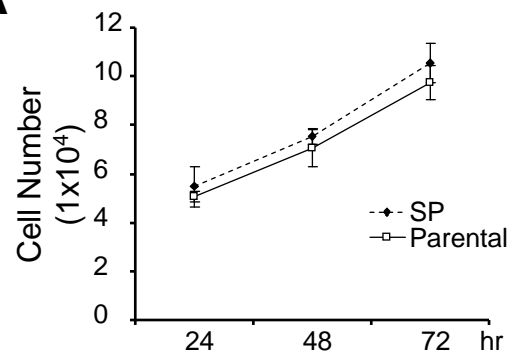**B**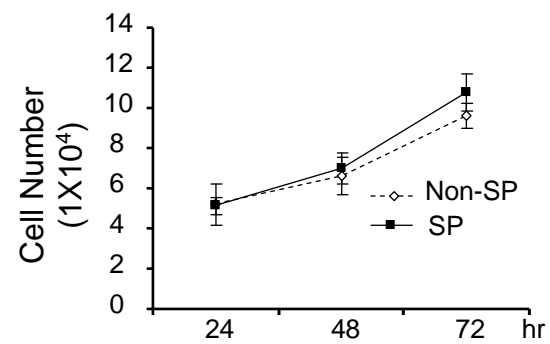**C**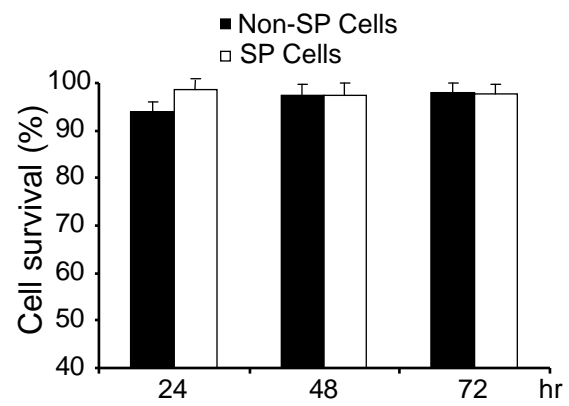

Supplement: Figure S1 — SP cells proliferate at the same rate as non-SP cells and parental HNSCC cells. (A): Both SP cells and parental HNSCC cells were grown in plates for 24, 48 and 72 hrs and cell numbers were counted. (B): Both SP and non-SP cells were grown in plates for 24, 48 and 72 hrs and cell numbers were counted. (C): Both SP and non-SP cells were cultured in plates for 24 hr and cell viability was determined. Afterwards, cells were re-cultured for additional 24 (48 hr time point) or 48 hr (72 hr time point) and cell viability was determined, respectively. (0.01 MB PDF) [file pone.0011456.s001.pdf]
